# Biomarker Profiles in Women with PCOS and PCOS Offspring; A Pilot Study

**DOI:** 10.1371/journal.pone.0165033

**Published:** 2016-11-02

**Authors:** Nadine M. P. Daan, Maria P. H. Koster, Marlieke A. de Wilde, Gerdien W. Dalmeijer, Annemieke M. V. Evelein, Bart C. J. M. Fauser, Wilco de Jager

**Affiliations:** 1 Department of Reproductive Medicine and Gynecology, University Medical Center Utrecht, Utrecht, the Netherlands; 2 Julius Centre for Health Sciences and Primary care, University Medical Center Utrecht, Utrecht, the Netherlands; 3 Laboratory of Translational Immunology, Division of Pediatrics, Wilhelmina Children's Hospital, Utrecht, the Netherlands; CHA University, REPUBLIC OF KOREA

## Abstract

**Objective:**

To study metabolic/inflammatory biomarker risk profiles in women with PCOS and PCOS offspring.

**Design:**

Cross-sectional comparison of serum biomarkers.

**Setting:**

University Medical Center Utrecht.

**Patients:**

Hyperandrogenic PCOS women (HA-PCOS, n = 34), normoandrogenic PCOS women (NA-PCOS, n = 34), non-PCOS reference population (n = 32), PCOS offspring (n = 14, age 6–8 years), and a paedriatic reference population (n = 30).

**Main Outcome Measure(s):**

Clustering profile of adipocytokines **(**IL-1b, IL-6, IL-13, IL-17, IL-18, TNF-α, adiponectin, adipsin, leptin, chemerin, resistin, RBP4, DPP-IV/sCD26, CCL2/MCP-1), growth factors **(**PIGF, VEGF, sVEGF-R1), soluble cell adhesion molecules **(**sICAM-1/sCD54, sVCAM-1/sCD106), and other inflammatory related proteases (MMP-9, S100A8, Cathepsin S). Differences in median biomarker concentrations between groups, and associations with the free androgen index (FAI; Testosterone/SHBG x100).

**Results:**

The cluster analysis identified leptin, RBP-4, DPP-IV and adiponectin as potential discriminative markers for HA-PCOS with a specifically strong correlation in cases with increased BMI. Leptin (R^2^ = 0.219) and adiponectin (R^2^ = 0.182) showed the strongest correlation with the FAI. When comparing median protein concentrations adult PCOS women with or without hyperandrogenemia, the most profound differences were observed for leptin (P < 0.001), DPP-IV (P = 0.005), and adiponectin (P < 0.001). Adjusting for age, BMI and multiple testing attenuated all differences. In PCOS offspring, MMP-9 (P = 0.001) and S100A8 (P < 0.001) concentrations were significantly higher compared to a healthy matched reference population, even after correcting for age and BMI and adjustment for multiple testing.

**Conclusion:**

In this preliminary investigation we observed significant differences in adipocytokines between women with or without hyperandrogenic PCOS and non-PCOS controls, mostly influenced by BMI. Leptin and adiponectin showed the strongest correlation with the FAI in adult women with PCOS. In PCOS offspring other inflammatory biomarkers (MMP-9, S100A8) were increased, suggesting that these children may exhibit increased chronic low-grade inflammation. Additional research is required to confirm results of the current exploratory investigation.

## Introduction

The polycystic ovary syndrome (PCOS) is the most common endocrinopathy amongst women of reproductive age, with a prevalence up to 15%.[1] PCOS is a heterogeneous condition of unknown origin, and is characterized by the presence of at least two of the following features: ovulatory dysfunction, hyperandrogenism, and polycystic ovarian morphology.[2] PCOS is associated with various cardiometabolic risk factors including obesity, hyperinsulinemia and dyslipidemia, which may result in type 2 diabetes mellitus, atherosclerosis and cardiovascular disease (CVD) later in life.[3–6] Moreover, PCOS is known to be associated with chronic low-grade inflammation as demonstrated by increased circulating inflammatory markers such as CRP, TNF-α, Il-18.[7–9]

Insulin resistance along with hyperandrogenism appear to play pivotal roles in the pathophysiology of PCOS.[10,11] Hyperandrogenism and obesity induce cardiometabolic dysfunction and chronic low-grade inflammation in women with PCOS.[12] The extent of increased cardiovascular events during later life in women with PCOS remains to be established. Children born to PCOS mothers may even exhibit increased cardiometabolic risk since early endocrine abnormalities (e.g. hyperandrogenism, hyperinsulinemia) and endothelial dysfunction have previously been reported in these children.[13,14]

Current research is therefore increasingly focusing on the discovery of novel biomarker profiles to further elucidate the complex pathophysiology of PCOS.[15,16] In the future, such biomarkers may serve to identify women with PCOS who are at particular risk for later life metabolic and cardiovascular disease, and who may benefit from secondary prevention strategies.

The current explorative study was designed to compare metabolic and inflammatory biomarker risk profiles between women with different PCOS phenotypes, offspring of PCOS mothers and reference populations. We hypothesized that metabolic and inflammatory biomarkers may be increased in women with PCOS and PCOS offspring at a young age. In doing so, we focussed on a wide range of tailored biomarkers based on the recent literature also including various closely-related novel biomarkers.

## Materials and Methods

Conduction of the current study was approved by the official Medical Ethical Committee Board of the University Medical Center Utrecht, and conducted according to the principles expressed in the Declaration of Helsinki. All included adult participants provided written informed consent, and written informed parental consent was obtained of all included children. Clinical trials were registered at www.clinicaltrials.gov, trial registration number NCT02309047 (adult PCOS), and NCT01767051 (PCOS offspring).

### Study population

#### PCOS

We included women with PCOS from a large prospective cohort study on menstrual cycle disturbances within the University Medical Center Utrecht. All women were evaluated through a standardized screening protocol which has been previously described in detail elsewhere.[5] PCOS was diagnosed according to the Rotterdam criteria in the presence of two or more of the following criteria: oligo‐ and/or anovulation, clinical and/or biochemical signs of hyperandrogenism, and polycystic ovarian morphology as assessed by transvaginal ultrasound.[2]

We included n = 34 hyperandrogenic women with PCOS (HA-PCOS), who exhibited ovulatory dysfunction, polycystic ovarian morphology and a free androgen index (FAI) > 4.5 [FAI: (Testosterone(nmol/L)/ SHBG(nmol/L)) x100)].[17] Subsequently we included n = 34 normoandrogenic women with PCOS (NA-PCOS) who exhibited ovulatory dysfunction, polycystic ovarian morphology, and a normal free androgen index (FAI) < 4.5. In this group only women with a normal body mass index (BMI) ≤25 kg/m^2^ were included, hence reflecting a truly mild PCOS reference group.

#### Non-PCOS reference population

We included n = 32 women without PCOS with regular menstrual cycles (21–35 days) from a cohort study regarding characteristics of women undergoing IVF/ICSI treatment within the University Medical Center Utrecht.All women included in the reference population were clinically evaluated and definitely classified as non-PCOS, hence composing a non-PCOS reference population. Serum samples were collected prior to the start of fertility treatment.

#### PCOS offspring

We included n = 14 children (6–8 years of age) who were born to PCOS mothers, from a cohort study regarding child health of children born to PCOS mothers within the University Medical Center Utrecht. Included children underwent a standardized screening with study procedures identical to those performed in the reference population (see below).[18,19]

*Pediatric reference population*. We included n = 30 children (7–8 years of age) from a population-based birth cohort study regarding determinants of wheezing illnesses and cardiovascular disease.[19] Included children underwent a standardized screening which has been described in detail elsewhere.[18,19]

Fasting serum samples were collected from all participants included in the current study. Serum samples were stored within 4 hours after withdrawal in -80 degrees Celsius.

### Multiplex immunoassay

Serum samples were used to measure the concentrations of 22 proteins, being: IL-1b, IL-6, IL-13, IL-17, IL-18, TNF-α, adiponectin, adipsin, leptin, chemerin, resistin, retinol-binding protein 4 (RBP4), dipeptidyl peptidase IV (DPP-IV/sCD26), monocyte chemotactic protein 1 (CCL2/MCP-1), placental growth factor (PIGF), vascular endothelial growth factor (VEGF), soluble VEGF receptor-1 (sVEGF-R1), soluble intercellular adhesion molecule 1 (sICAM-1/sCD54), soluble vascular cell adhesion molecule 1 (sVCAM-1/sCD106), matrix metallopeptidase 9 (MMP-9), S100A8, Cathepsin S.

Laboratory measurements were performed using an in-house developed and validated multiplex immunoassay based on Luminex technology (xMAP, Luminex Austin TX USA). The assay was performed using previously described methods.[20–22] In short, a-specific heterophilic immunoglobulins were preabsorbed from all samples with heteroblock (Omega Biologicals, Bozeman MT, USA). Then samples were incubated with antibody-conjugated MagPlex microspheres for one hour at room temperature with continuous shaking, followed by one hour incubation with biotinylated antibodies, and 10 min incubation with phycoerythrin-conjugated streptavidin diluted in high performance ELISA buffer (HPE, Sanquin the Netherlands). Data acquisition was performed with the Biorad FlexMAP3D (Biorad laboratories, Hercules USA) in combination with xPONENT software version 4.2 (Luminex). Laboratory data were analyzed by 5-parametric curve fitting using Bio-Plex Manager software, version 6.1.1 (Biorad).

### Statistical analyses

Study sample size was determined according to availability of serum samples. Basic descriptive statistics were used to describe the patient population.

Kruskal Wallis tests were performed to compare patient characteristics and one-way ANOVA were performed to compare log-transformed biomarker values between HA-PCOS women, NA-PCOS women and non-PCOS women. When this resulted in a P-value < 0.05, pairwise Student’s T-test were used to calculate P-values between specific groups. Next, P-values were adjusted for age and BMI using general linear models, and false discovery rates (FDR) were calculated to correct for multiple testing. In the pediatric populations, Mann-Whitney U tests and Chi-square tests were used to compare baseline characteristics. Student’s t-tests were used to assess differences in log-transformed biomarker concentrations between both groups, including specific sub-analyses for gender. P-values were adjusted for age and BMI using general linear models and FDR was calculated to correct for multiple testing.

Before log-transformation, biomarker values that were below the lower limit of detection were imputed at 35% of the lower detection limit concentration. Values which were above the highest limit of detection were imputed as the highest value of detection concentration plus one pg/ml.

Finally, as described previously, an unsupervised hierarchal clustering analysis, with min-max normalization per protein, was performed to investigate the discriminative potential of a single or a combination of proteins [23].

All statistical analyses were performed with SPSS Statistics 21.0. Hierarchical cluster analyses were performed using Omniviz 6.1.2 (Instem Scientific).

## Results

First, we assessed whether protein concentrations differ between HA-PCOS, NA-PCOS and the non-PCOS reference group. Clinical baseline characteristics of involved study groups are shown in Table 1.

**Table 1:** Baseline characteristics of women with PCOS and non PCOS reference group.

		HA-PCOS (n = 34)	NA-PCOS (n = 34)	non-PCOS (n = 32)	P-value
**Ethnicity**					0.54
	Caucasian	31 (91)	33 (97)	28 (90)	
	Mediterranean	1 (3)	-	2 (7)	
	Indian	2 (6)	-	-	
	Black	-	1 (3)	-	
	Asian	-	-	1 (3)	
**Age** (years)		28.5 [23.5–32.5]	28.8 [25.8–31.2]	34.5 [30.7–37.7]	< 0.001 ^a^^,^^c^
**FAI**		6.7 [5.0–10.2]	2.0 [1.4–2.6]	-	
**BMI** (kg/m^2^)		29.5 [23.3–35.6]	21.8 [19.8–22.2]	22.5 [21.2–24.5]	< 0.001^a^^,^^b^^,^^c^
**ART indication**					-
	Idiopathic infertility	-	-	13 (41)	
	Male factor	-	-	15 (47)	
	Tubal factor	-	-	2 (6)	
	Low ovarian reserve	-	-	1 (3)	
	Donor semen	-	-	1 (3)	

PCOS: polycystic ovary syndrome, NA: normoandrogenic, HA: hyperandrogenic, FAI: free androgen index (Testosterone/SHBG)x100), BMI: body mass index, ART: assisted reproduction technology. Values are depicted as medians [interquartile ranges], or absolute numbers (percentages). Depicted variables contained a maximum of 3% missing values. P-values were calculated using Kruskal Wallis ANOVA. When a P-value < 0.05 was detected pairwise Mann Whitney U tests were used to assess differences between specific groups.

^a^ P < 0.05 between HA PCOS and non PCOS.

^b^ P < 0.05 between HA PCOS and NA PCOS.

^c^ P < 0.05 between NA PCOS and non PCOS

**Fig 1:**
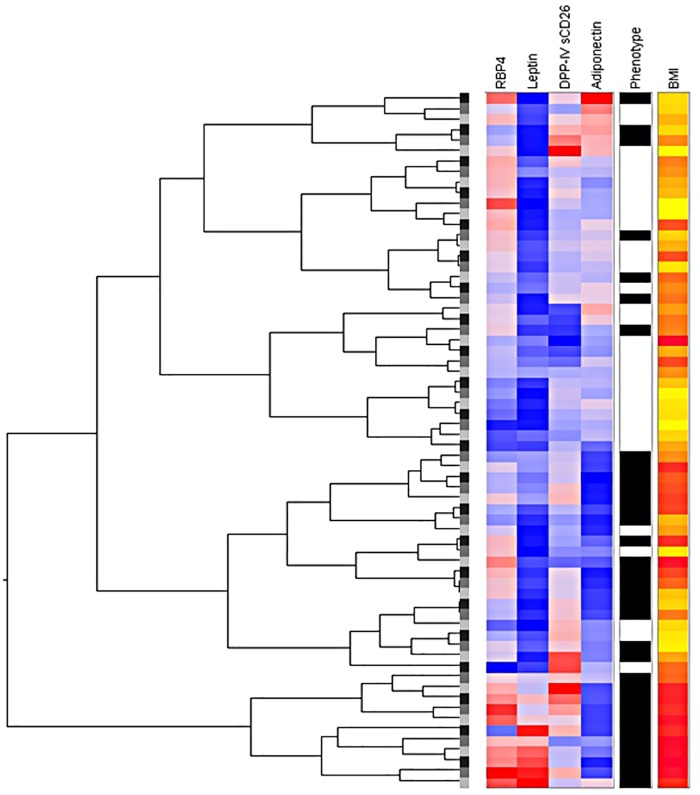
Hierarchical cluster analysis in women with hyperandrogenic PCOS and non-PCOS women. PCOS: polycystic ovary syndrome, RBP-4: retinol-binding protein 4, DPP-IV/sCD26: dipeptidyl peptidase IV. Phenotype: black represents hyperandrogenic PCOS; White represents non-PCOS reference population. BMI: yellow represents low BMI, red represents high BMI.

In order to assess the discriminative potential of a single or a combination of any marker(s) in HA-PCOS, NA-PCOS and the non-PCOS group, we performed a hierarchical cluster analysis as described by van den Ham et al.[23] This analysis showed clustering of leptin, RBP-4, DPP-IV and adiponectin specific for HA-PCOS with a strong correlation of cases with increased BMI, as is depicted in Fig 1. The complete clustering of all proteins is shown in S1 Fig.

**Fig 2:**
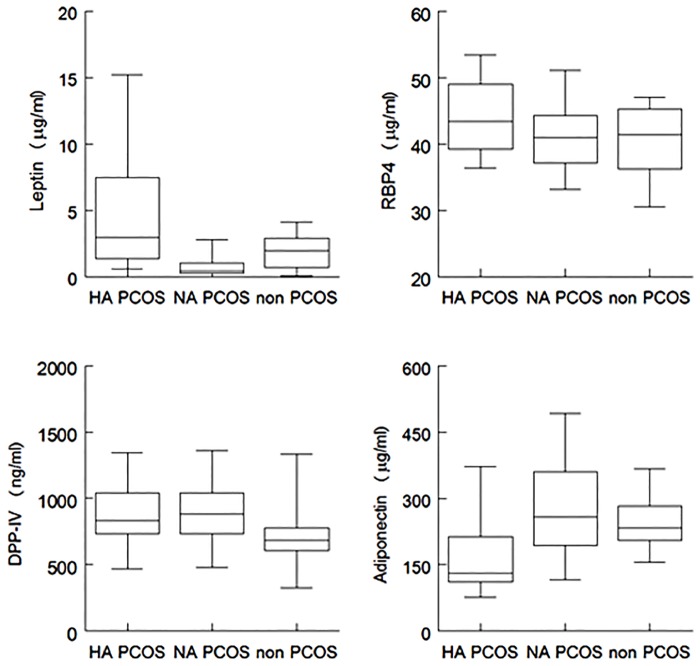
Biomarker concentrations of most discriminative markers in women with PCOS and non-PCOS women. RBP4: retinol-binding protein 4, DPP-IV/sCD26: dipeptidyl peptidase IV. PCOS: polycystic ovary syndrome, HA: hyperandrogenic, NA: normoandrogenic, IL: interleukin,, DPP-IV/sCD26: dipeptidyl peptidase IV.

Fig 2 shows the discriminative potential of these four markers in both PCOS groups and the non-PCOS group.

**Fig 3:**
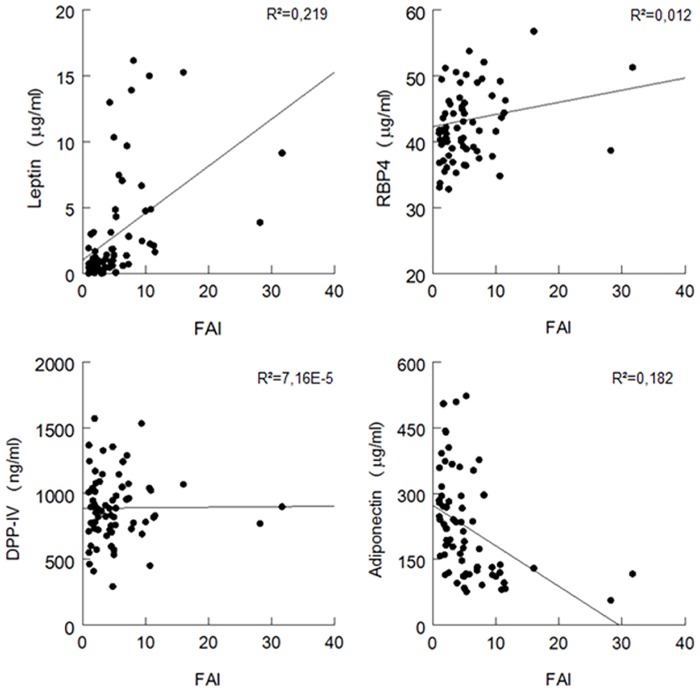
Correlation between biomarker concentrations and the free androgen index (FAI) in women with PCOS. RBP-4: retinol-binding protein 4, DPP-IV/sCD26: dipeptidyl peptidase IV.

Subsequently, we assessed the correlation between these four potential biomarkers and the FAI, being one of the clinical features of PCOS. The strongest correlations were found for leptin (R^2^ = 0.219) and adiponectin (R^2^ = 0.182) as shown in, Fig 3.

Next, we compared differences in median protein concentrations between adult PCOS groups (see Tables 2 and 3). The most profound differences were found for leptin (P <0.001), DPP-IV (P = 0.005), and adiponectin (P < 0.001). Differences in leptin and adiponectin concentrations between groups were no longer significant after adjustment for BMI and age. DPP-IV remained significantly increased in HA-PCOS versus non-PCOS women after adjustment for BMI and age (P = 0.049), however significance was last after correction for multiple testing (P = 0.29).

**Table 2:** Biomarker concentrations in PCOS women and non-PCOS reference group.

	HA-PCOS (n = 34)	NA-PCOS (n = 34)	non-PCOS (n = 32)	P-value
**IL-13** (pg/ml)	4.5 [0.9–19.5]	5.4 [1.5–29.2]	0.9 [0.9–6.6]	**0.043**
**IL-18** (pg/ml)	95 [52–135]	75 [47–103]	87 [61–116]	0.53
**CCL2/MCP-1** (pg/ml)	101 [83–139]	111 [82–136]	112 [80–152]	0.25
**PIGF** (pg/ml)	35 [18–46]	28 [14–42]	28 [15–44]	0.33
**VEGF** (ng/ml)	0.4 [0.3–0.5]	0.3 [0.1–0.5]	0.4 [0.2–0.7]	0.34
**MMP-9** (μg/ml)	1.8 [1.2–3.1]	1.3 [0.8–2.2]	1.7 [0.8–2.4]	0.16
**sVEGF-R1** (ng/ml)	1.4 [1.2–1.7]	1.5 [1.4–1.9]	1.4 [1.1–1.6]	0.053
**S100A8** (ng/ml)	2.5 [1.2–4.5]	0.9 [0.3–4.6]	2.6 [0.7–5.3]	0.19
**Adipsin** (ng/ml)	0.4 [0.2–0.4]	0.3 [0.2–0.4]	0.3 [0.2–0.4]	0.31
**Leptin** (ng/ml)	3.0 [1.4–7.9]	0.4 [0.3–1.1]	1.9 [0.7–2.9]	**<0.001**
**Resistin** (ng/ml)	38 [27–54]	31 [24–39]	35 [30–45]	**0.017**
**RBP4** (μg/ml)	43 [39–49]	41 [37–44]	41 [36–45]	0.10
**DPP-IV/ sCD26** (μg/ml)	0.8 [0.7–1.0]	0.9 [0.7–1.0]	0.7 [0.6–0.8]	**0.005**
**sICAM1/ sCD54**(μg/ml)	0.3 [0.3–0.4]	0.3 [0.3–0.4]	0.3 [0.3–0.4]	0.97
**sVCAM** (μg/ml)	3.7 [3.2–4.5]	3.8 [3.2–4.5]	4.0 [3.2–5.1]	0.42
**Cathepsin S** (ng/ml)	13 [10–15]	11 [9–12]	11 [10–14]	**0.009**
**Adiponectin** (μg/ml)	131 [111–219]	258 [192–362]	233 [204–285]	**<0.001**

Values represent median concentrations [interquartile ranges]. P-values were calculated with ANOVA on logtransformed values for difference between all groups. PCOS: polycystic ovary syndrome, HA: hyperandrogenic, NA: normoandrogenic, IL: interleukin, CCL2/MCP-1: monocyte chemoattractant protein-1, PIGF: placental growth factor, VEGF: vascular endothelial growth factor, MMP-9: matrix metallopeptidase 9, RBP-4: retinol-binding protein 4, DPP-IV/sCD26: dipeptidyl peptidase IV, sICAM: soluble intercellular adhesion molecule 1, sVCAM: soluble vascular cell adhesion molecule 1. IL-1b, IL-6, IL-17, TNF-α and chemerin are not shown as the majority of samples (>57%) were undetectable measurements evenly distributed amongst the study population.

**Table 3:** P-values, after adjustment for BMI and Age, and correction for multiple testing (FDR).

	ANOVA all groups	HA-PCOS vs non-PCOS			HA-PCOS vs NA-PCOS			NA-PCOS vs non-PCOS		
	P-value	P-value	adjustedP-value	FDR	P-value	Adjusted P-value	FDR	P-value	Adjusted P-value	FDR
**IL-13** (pg/ml)	**0.043**	0.10	0.30	0.45	0.39	0.83	0.91	0.013	0.46	0.92
**Leptin** (ng/ml)	**<0.001**	0.006	0.29	0.45	<0.001	0.08	0.24	0.013	0.08	0.48
**Resistin** (ng/ml)	**0.017**	0.72	0.42	0.50	0.010	0.40	0.60	0.021	0.92	0.92
**DPP-IV/sCD26** (μg/ml)	**0.005**	0.014	0.049	0.29	0.70	0.91	0.91	0.005	0.48	0.92
**Cathepsin S** (ng/ml)	**0.009**	0.85	0.67	0.67	0.005	0.20	0.40	0.012	0.65	0.92
**Adiponectin** (μg/ml)	**<0.001**	<0.001	0.15	0.45	<0.001	0.07	0.24	0.38	0.85	0.92

When a P-value < 0.05 was detected in ANOVA, pairwise T-test on logtransformed biomarkers were used to calculate P-values between specific groups. Furthermore P-values were adjusted for age and BMI, and false discovery rates (FDR) were calculated to correct for multiple testing. PCOS: polycystic ovary syndrome, HA: hyperandrogenic, NA: normoandrogenic, IL: interleukin,, DPP-IV/sCD26: dipeptidyl peptidase IV.

Since such shifted protein profiles as well as certain clinical characteristics (e.g. BMI) are known to be associated with a predisposition for cardiovascular disease, we assessed the identical protein panel in a cross sectional cohort of PCOS offspring and paediatric reference cohort of comparable age and sex, based on the assumption that PCOS offspring may already exhibit an increased cardiometabolic risk at young age. The baseline characteristics of these two groups are shown in Table 4. Results of the hierarchical cluster analysis in these groups are shown in S2 Fig When comparing protein concentrations between PCOS offspring and the reference population, we found higher MMP-9 (*P* = 0.001) and S100A8 (*P* < 0.001) concentrations in the PCOS offspring (see Table 5). Correction for BMI and age yielded similar results. and differences remained significant after correction for multiple testingSubanalyses regarding the potential influence of gender, comparing both sexes separately, showed that the significant difference in MMP-9 concentrations was mainly driven by the difference in girls (girls 1.0 μg/ml [0.7–2.0] vs. 0.2 μg/ml [0.1–0.5], boys 0.8 μg/ml [0.4–2.2] vs. 0.3 μg/ml [0.2–0.5]). Differences in S100A8 concentrations were significant in both sexes (girls 2.7 ng/ml [0.8–2.8] vs. 0.0 ng/ml [0.0–0.8], boys 2.2 ng/ml [0.2–3.7] vs. 0.0 ng/ml [0.0–0.0]).

**Table 4:** Baseline characteristics of PCOS offspring and reference group.

		PCOS offspring (n = 14)	Reference group (n = 30)	P-value
**Complications during pregnancy**				0.08
	None	8 (57)	25 (86)	
	Hypertensive complications	5 (36)	2 (7)	
	Gestational diabetes	1 (7)	-	
	Infection	1 (7)	2 (7)	
**Mode of delivery**				0.05
	Vaginal spontaneously	7 (50)	23 (79)	
	Caesarean section	5 (36)	6 (21)	
	Assisted vaginal delivery	2 (14)	-	
**Gestational age at delivery** (weeks)		40.1 [37.5–40.4]	40.4 [39.4–41.2]	0.19
**Preterm delivery**				0.15
	Yes	1 (7)	-	
	No	13 (93)	29 (100)	
**Birth Weight** (grams)		3295 [3061–3651]	3600 [3200–3940]	0.27
**Neonatal complications**				
	Small for gestational age	1 (7)	1 (3)	0.15
	Large for gestational age	2 (14)	2 (7)	0.43
**Sex**				0.98
	Male	6 (43)	13 (43)	
	Female	8 (57)	17 (57)	
**Age at screening** (years)		7.0 [6.6–8.1]	7.8 [7.6–7.9]	0.09
**BMI** (kg/m^2^)		15.7 [14.5–16.8]	14.9 [14.5–16.0]	0.12

Values represent median values [interquartile range] or absolute numbers (percentages) Mann-Whitney U tests or Chi-square tests were used to calculate P-values. Variables contained a maximum of 3% missing values.

**Table 5:** Median biomarker concentrations in PCOS offspring and reference group.

	PCOS offspring (n = 14)	Reference group (n = 30)	P-value	Adjusted P-value	FDR
**IL-13** (pg/ml)	8.5 [0.9–37.7]	16.5 [5.0–77.6]	0.14	0.06	0.26
**IL-18** (pg/ml)	134 [85–196]	139 [107–188]	0.64	0.73	0.78
**CCL2/MCP-1** (pg/ml)	113 [88–138.]	134 [100–159]	0.18	0.22	0.47
**PIGF** (pg/ml)	37 [32–59]	31 [16–69]	0.17	0.29	0.53
**VEGF** (ng/ml)	0.3 [0.1–0.6]	0.2 [0.1–0.5]	0.59	0.65	0.74
**MMP-9** (μg/ml)	1.0 [0.6–2.2]	0.2 [0.2–0.5]	**0.001**	**0.003**	**0.02**
**sVEGF-R1** (ng/ml)	1.4 [1.1–1.7]	1.6 [1.1–2.0]	0.47	0.46	0.71
**S100A8** (ng/ml)	2.7 [0.7–3.1]	0.0 [0.0–0.1]	**<0.001**	**<0.001**	**<0.001**
**Adipsin** (ng/ml)	0.3 [0.1–0.3]	0.3 [0.2–0.3]	0.71	0.96	0.96
**Leptin** (ng/ml)	0.1 [0.0–0.5]	0.0 [0.0–0.3]	0.31	0.63	0.74
**Resistin** (ng/ml)	26 [21–32]	22 [19–36]	0.53	0.18	0.44
**RBP4** (μg/ml)	35 [32–37]	35 [32–40]	0.20	0.05	0.26
**DPP-IV/sCD26** (μg/ml)	2.2 [1.8–2.7]	2.5 [2.0–3.1]	0.19	0.08	0.27
**sICAM1/sCD54**(μg/ml)	0.5[0.4–0.6]	0.5 [0.4–0.6]	0.50	0.50	0.71
**sVCAM** (μg/ml)	7.7 [6.5–10.3]	6.9 [5.7–9.4]	0.18	0.16	0.44
**Cathepsin S** (ng/ml)	12 [10–13]	11 [11–13]	0.42	0.31	0.53
**Adiponectin** (μg/ml)	273 [215–312]	258 [239–325]	0.70	0.61	0.74

Values represent median concentrations [interquartile ranges]. P-values were calculated using T-tests on logtransformed biomarkers. Furthermore P-values were adjusted for age and BMI (general linear models), and false discovery rates (FDR) were calculated to correct for multiple testing. PCOS: polycystic ovary syndrome, CCL2/MCP-1: monocyte chemoattractant protein-1, PIGF: placental growth factor, VEGF: vascular endothelial growth factor, MMP-9: matrix metallopeptidase 9, RBP-4: retinol-binding protein 4, DPP-IV/sCD26: dipeptidyl peptidase IV, sICAM1/sCD54: soluble intercellular adhesion molecule 1, sVCAM: soluble vascular cell adhesion molecule 1. IL-1b, IL-6, IL-17, TNF-α and chemerin are not shown as the majority of samples (>57%) were undetectable measurements evenly distributed amongst the study population.

## Discussion

The primary aim of this study was to compare metabolic/inflammatory biomarker risk profiles between women with different PCOS phenotypes, PCOS offspring and reference populations. We selected specific adipocytokines**,** growth factors**,** soluble cell adhesion molecules**,** and other proteins, which have previously been associated with chronic low-grade inflammation and cardiometabolic dysfunction.

Adipocytokines are known to affect vascular endothelium by stimulating the migration of monocytes into the vessel wall, and inducing the conversion of monocytes into macrophages.[24] These macrophages phagocytize LDL cholesterol and form fatty streaks within the vessel wall, ultimately resulting in atherosclerotic plaque formation.[25] Adipocytokines, such as IL-6 and TNF-alpha, may be valuable cardiovascular risk markers in women with PCOS, since they reflect chronic low-grade inflammation and have been associated with several insulin-resistant states.[24] Furthermore, vascular endothelial growth factors may stimulate the development of atherosclerosis and/or plaque instability through the formation of microvessels.[26] Increased expression of cell adhesion molecules on the vascular endothelium may play a significant role in atherosclerosis as well, by inducing solid adhesion of inflammatory cells to the vascular surface.[27] Hence these biomarkers were specifically selected to differentiate risk potential within PCOS, in view of the future appliance of secondary prevention measures.

When comparing women with PCOS to a non-PCOS reference population, we found significant differences in several adipocytokines, which were mostly influenced by BMI. Leptin and adiponectin appeared the most discriminative markers in women with PCOS, both showing the strongest correlation with the FAI. This finding corroborates with previous studies in which these adipocytokines were correlated with metabolic complications in women with PCOS.[28,29] Leptin is secreted by white adipose tissue and regulates food intake and energy expenditure through actions on the central nervous system.[30] Serum concentrations rise with increasing body weight and impair insulin activity, and may therefore attribute to the development of hyperandrogenism and infertility in PCOS.[31] High concentrations may also directly impair ovarian function by decreasing ovarian sensitivity for gonadotrophins.[32] Previous reports concerning leptin concentrations in PCOS and non-PCOS women have not been entirely consistent, although the majority of studies report no difference in leptin concentrations when comparing PCOS women to weight-matched non-PCOS controls, which is in line with our findings in the current study.[33–35]

Adiponectin is secreted in adipose tissue and exerts anti-atherogenic, anti-inflammatory and insulin-sensitizing effects.[36] It has been shown that serum concentrations of this protein are inversely correlated to obesity, insulin resistance and diabetes mellitus type 2.[36,37] Circulating concentrations appear to be lower in women with PCOS compared to non-PCOS women, even after adjustment for BMI-related effects.[38]

We observed no significant differences in other inflammatory biomarkers between women with and without PCOS (e.g. interleukins, TNF-α, resistin, MMP-2, MMP-9, MCP-1), as opposed to what has previously been reported by others.[7,8,39–41] This may be due to the relatively low BMI of women with PCOS included in the current study, even in the overweight HA-PCOS group in which the average BMI did not exert 30 kg/m^2^. [24]

Contrary to adult PCOS, we did observe significantly higher concentrations of MMP-9 and S100A8 in PCOS offspring, compared to the reference population. Increased circulating MMP-9 concentrations have been previously reported in adult women with PCOS.[40,42] MMP-9 is a zinc-binding proteolytic enzyme which is involved in remodelling of the extracellular matrix.[40] It originates from various inflammatory cells, however predominantly from neutrophils.[43] Within the ovary, MMPs are involved in follicular development and the process of ovulation.[42] It has been hypothesized that increased MMP-9 concentrations may be associated with the pathophysiology of PCOS, since altered extracellular matrix remodeling may be related to inappropriate follicular atresia and increased ovarian stroma tissue.[40,44]. Increased MMP activity has been associated with various other disease processes including asthma, cystic fibrosis, ulcerative colitis, atherosclerosis and cardiovascular disease. [45–49]

To our knowledge, the possible role of circulating S100A8 in women with PCOS or PCOS offspring has not been previously addressed. S100A8 is known as a damage-associated molecular pattern (DAMP) molecule because of its pro-inflammatory actions, and is secreted in response to cell damage, death, and stress.[50] Moreover, this chemotaxin is involved in various processes including calcium homeostasis, cellular migration, and energy metabolism.[51] Within the ovary, S100A8 might also play a role in primordial follicle formation, as was recently demonstrated in a fetal mice model.[52]

In PCOS offspring we did not observe a difference in adipocytokines compared to the reference group. This might be due to an absence of overweight and insulin resistance in these young prepuberal children. As children enter puberty, insulin resistance may develop in response to growth hormone secretion which induces accelerated growth during puberty.[53] It is known that insulin acts as a co-gonadotropin in ovarian steroidogenesis and as such may contribute to the development of PCOS.[11]

To our knowledge, this is the first study in which metabolic/inflammatory biomarkers were simultaneously assessed both in adult women with PCOS as well as PCOS offspring. Further strengths of this study are the large tailored series of potentially relevant biomarkers which were assessed in a well-phenotyped patient population, and the use of a validated multiplex immunoassay with standardized technology which has been repeatedly described before.[20–22]

Limitations of the current study are the relatively small sample size, and the characteristics of the reference populations. The current study was performed with a relatively limited sample size, especially concerning PCOS offspring. Therefore, reported results in these children may be regarded as preliminary and require validation in a larger cohort. The adult reference population consisted of women who were planned to undergo IVF/ICSI treatment, and therefore might not be considered as entirely healthy controls despite the fact that these women had regular menstrual cycles. However, post-hoc analyses including only women with infertility due to a male factor did not significantly alter results (data not shown). Moreover, all women included in the adult reference population were clinically evaluated and definitely classified as non-PCOS. Although the paediatric reference population was clinically well defined, there was no detailed reproductive history data available of their mothers and therefore PCOS could not be excluded. Hence, it is possible that observed differences in protein concentrations between PCOS offspring and the reference group in the current study may be underestimated.

In summary, we observed differences in adipocytokines between women with normoandrogenic and hyperandrogenic PCOS and women without PCOS, which were influenced by BMI. Leptin and adiponectin showed the strongest correlation with the FAI. Since these biomarkers are directly correlated with more easy assessable classical risk factors such as obesity and insulin resistance, routine assessment of these markers at this point may contribute little to the conventional risk assessment in women with PCOS.

In PCOS offspring other inflammatory biomarkers were higher, suggesting that these young children may exhibit increased risk of chronic low-grade inflammation. Although these results require validation in a larger cohort study, these findings may be of importance for future appliance of primary prevention measures. Foremost,longitudinal follow-up studies with repeated measurements are needed in order to assess the potential association between biomarker profiles, the development of PCOS and actual cardiovascular disease in later life.

## Supporting Information

S1 Fig Hierarchical cluster analysis in women with hyperandrogenic PCOS and non-PCOS women. PCOS: polycystic ovary syndrome, IL: interleukin, CCL2/MCP-1: monocyte chemoattractant protein-1, PIGF: placental growth factor, VEGF: vascular endothelial growth factor, MMP-9: matrix metallopeptidase 9, RBP-4: retinol-binding protein 4, DPP-IV/sCD26: dipeptidyl peptidase IV, sICAM: soluble intercellular adhesion molecule 1, sVCAM: soluble vascular cell adhesion molecule 1. IL-1b, IL-6, IL-17, TNF-α and chemerin are not shown as the majority of samples (>57%) were undetectable measurements evenly distributed amongst the study population. Phenotype: black represents hyperandrogenic PCOS; White represents non-PCOS reference population. BMI: yellow represents low BMI, red represents high BMI.
(TIF)

S2 Fig Hierarchical cluster analysis in PCOS offspring and reference population. PCOS: polycystic ovary syndrome, IL: interleukin, CCL2/MCP-1: monocyte chemoattractant protein-1, PIGF: placental growth factor, VEGF: vascular endothelial growth factor, MMP-9: matrix metallopeptidase 9, RBP-4: retinol-binding protein 4, DPP-IV/sCD26: dipeptidyl peptidase IV, sICAM: soluble intercellular adhesion molecule 1, sVCAM: soluble vascular cell adhesion molecule 1. IL-6, and chemerin are not shown as > 95% were undetectable measurements evenly distributed amongst the study population. Black represents PCOS offspring, white represents reference population.
(TIF)
