# Supplementary figures and images for: Biomarker Profiles in Women with PCOS and PCOS Offspring; A Pilot Study
Source: PLoS One. 2016 Nov 2;11(11):e0165033. doi: 10.1371/journal.pone.0165033 (PMC5091782; doi:10.1371/journal.pone.0165033)

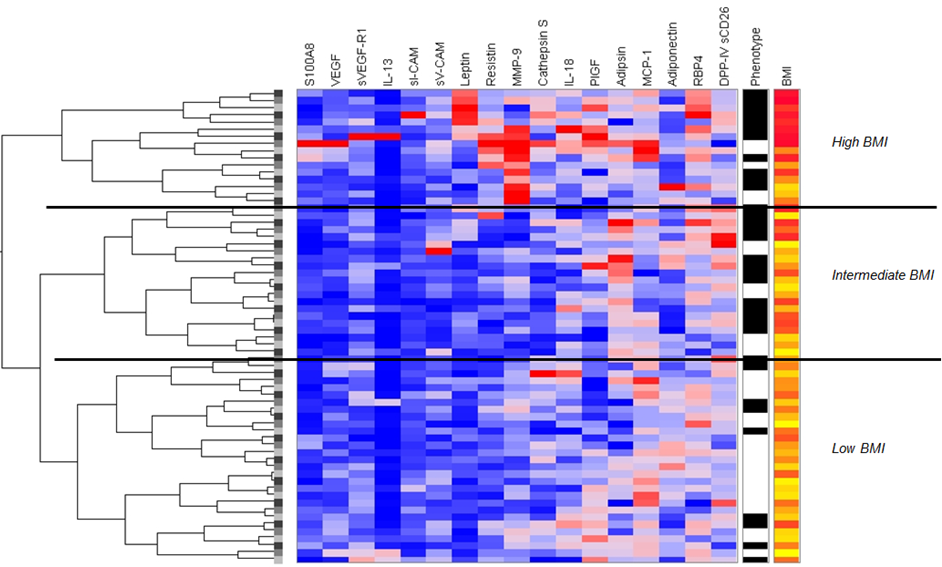

Supplement: S1 Fig — PCOS: polycystic ovary syndrome, IL: interleukin, CCL2/MCP-1: monocyte chemoattractant protein-1, PIGF: placental growth factor, VEGF: vascular endothelial growth factor, MMP-9: matrix metallopeptidase 9, RBP-4: retinol-binding protein 4, DPP-IV/sCD26: dipeptidyl peptidase IV, sICAM: soluble intercellular adhesion molecule 1, sVCAM: soluble vascular cell adhesion molecule 1. IL-1b, IL-6, IL-17, TNF-α and chemerin are not shown as the majority of samples (>57%) were undetectable measurements evenly distributed amongst the study population. Phenotype: black represents hyperandrogenic PCOS; White represents non-PCOS reference population. BMI: yellow represents low BMI, red represents high BMI. (TIF) [file pone.0165033.s001.tif]

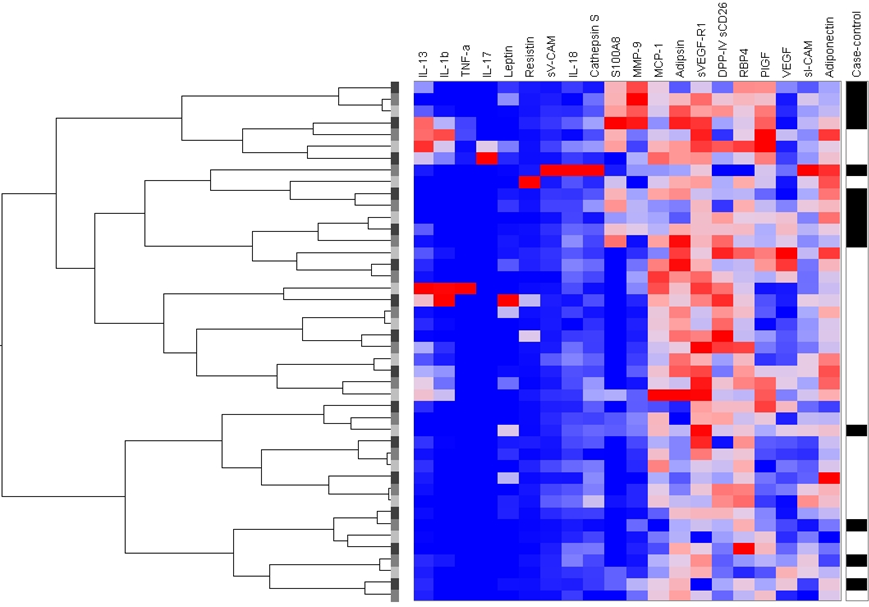

Supplement: S2 Fig — PCOS: polycystic ovary syndrome, IL: interleukin, CCL2/MCP-1: monocyte chemoattractant protein-1, PIGF: placental growth factor, VEGF: vascular endothelial growth factor, MMP-9: matrix metallopeptidase 9, RBP-4: retinol-binding protein 4, DPP-IV/sCD26: dipeptidyl peptidase IV, sICAM: soluble intercellular adhesion molecule 1, sVCAM: soluble vascular cell adhesion molecule 1. IL-6, and chemerin are not shown as > 95% were undetectable measurements evenly distributed amongst the study population. Black represents PCOS offspring, white represents reference population. (TIF) [file pone.0165033.s002.tif]
